# Supplementary figures and images for: Glutamine metabolism modulates chondrocyte inflammatory response
Source: eLife. 2022 Aug 2;11:e80725. doi: 10.7554/eLife.80725 (PMC9371604; doi:10.7554/eLife.80725)

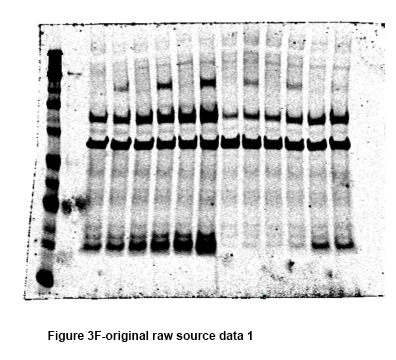

Supplement: Figure 3—source data 2. [file elife-80725-fig3-data2.zip › Figure 3F-original raw source data 1.tif]

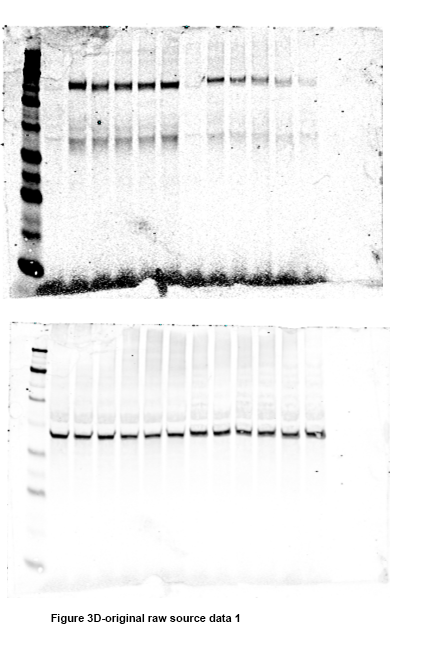

Supplement: Figure 3—source data 2. [file elife-80725-fig3-data2.zip › Figure 3D-original raw source data 1.tif]

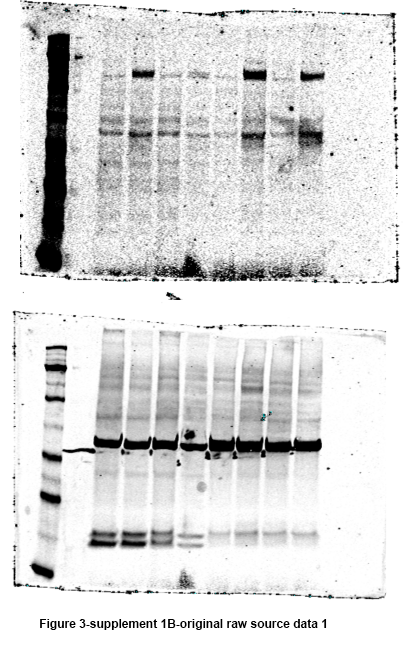

Supplement: Figure 3—figure supplement 1—source data 2. [file elife-80725-fig3-figsupp1-data2.zip › Figure 3-supplement 1B-original raw source data 1B.tif]

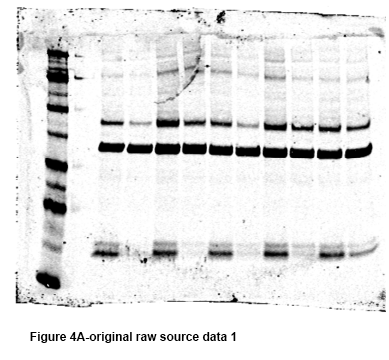

Supplement: Figure 4—source data 2. [file elife-80725-fig4-data2.zip › Figure 4A-original raw source data 1.tif]

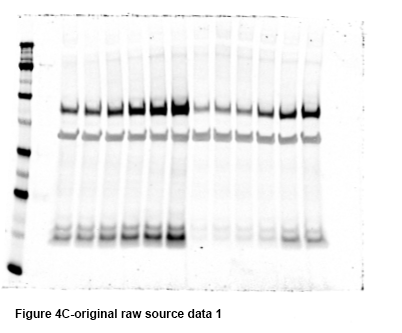

Supplement: Figure 4—source data 2. [file elife-80725-fig4-data2.zip › Figure 4C-original raw source data 1.tif]

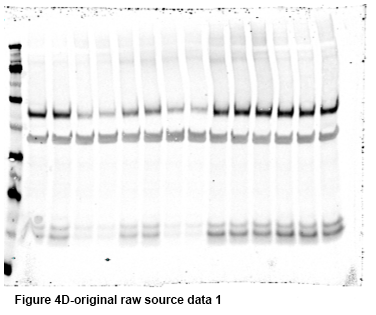

Supplement: Figure 4—source data 2. [file elife-80725-fig4-data2.zip › Figure 4D-original raw source data 1.tif]

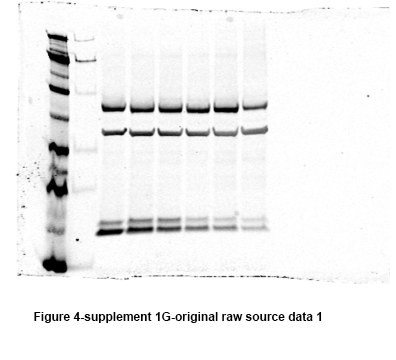

Supplement: Figure 4—figure supplement 1—source data 2. [file elife-80725-fig4-figsupp1-data2.zip › Figure 4-supplement 1G-original raw source data 1.tif]

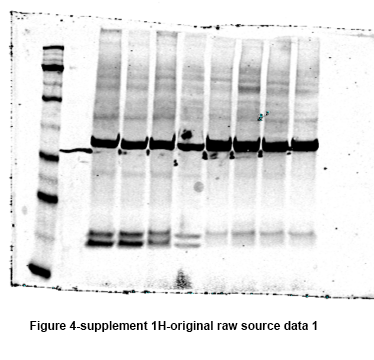

Supplement: Figure 4—figure supplement 1—source data 2. [file elife-80725-fig4-figsupp1-data2.zip › Figure 4-supplement 1H-original raw source data 1.tif]

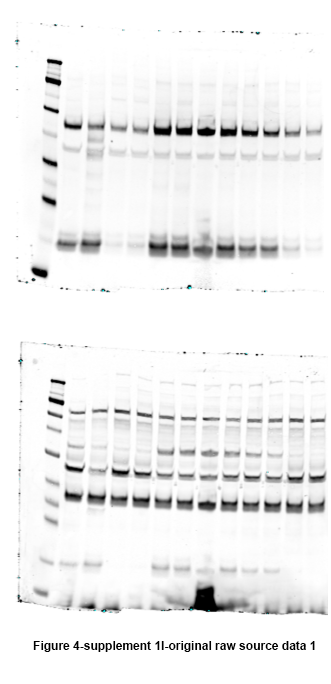

Supplement: Figure 4—figure supplement 1—source data 2. [file elife-80725-fig4-figsupp1-data2.zip › Figure 4-supplement 1I-original raw source data 1.tif]

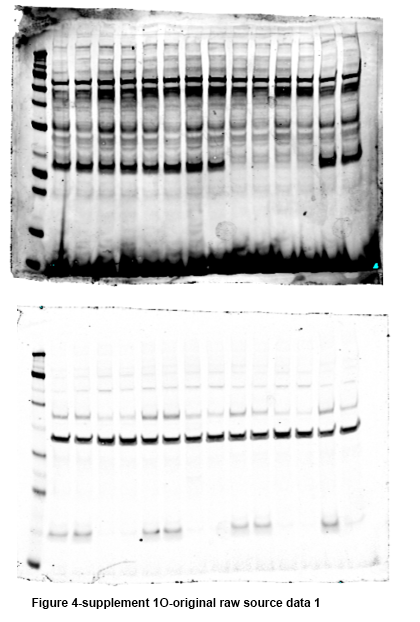

Supplement: Figure 4—figure supplement 1—source data 2. [file elife-80725-fig4-figsupp1-data2.zip › Figure 4-supplement 1O-original raw source data 1.tif]

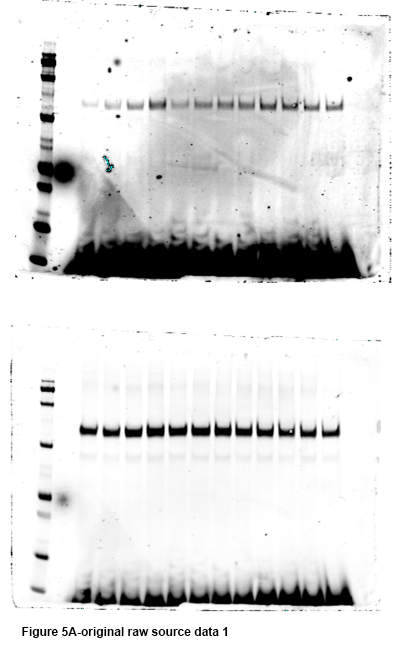

Supplement: Figure 5—source data 2. [file elife-80725-fig5-data2.zip › Figure 5A-original raw source data 1.tif]

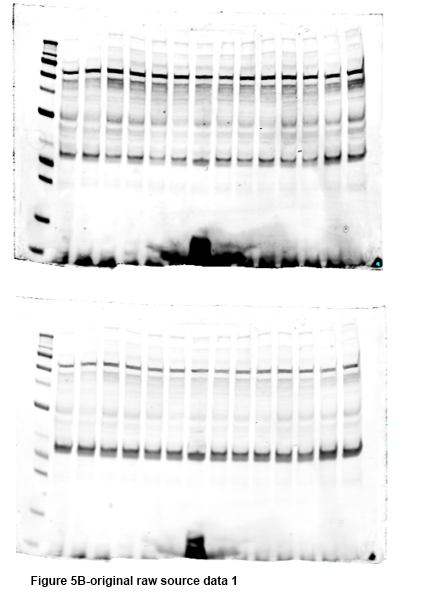

Supplement: Figure 5—source data 2. [file elife-80725-fig5-data2.zip › Figure 5B-origianl raw source data 1.tif]

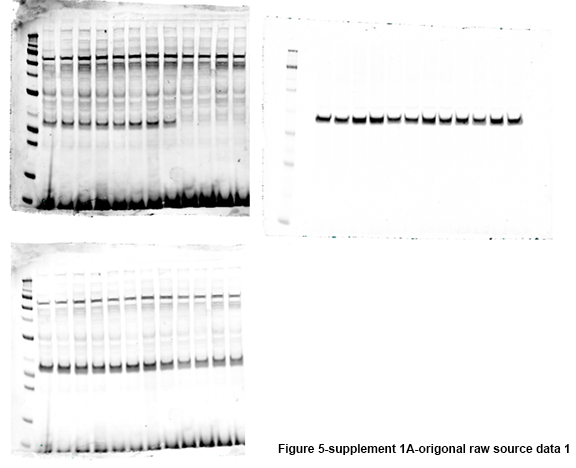

Supplement: Figure 5—figure supplement 1—source data 2. [file elife-80725-fig5-figsupp1-data2.zip › Figure 5-supplement 1A-original raw source data 1.tif]
